# Variation in Inflammatory Response during Pneumococcal Infection Is Influenced by Host-Pathogen Interactions but Associated with Animal Survival

**DOI:** 10.1128/IAI.01057-15

**Published:** 2016-03-24

**Authors:** Magda S. Jonczyk, Laura Escudero, Nicolas Sylvius, Martin Norman, Birgitta Henriques-Normark, Peter W. Andrew

**Affiliations:** a Department of Infection, Immunity and Inflammation, University of Leicester, Leicester, United Kingdom; b Department of Genetics, University of Leicester, Leicester, United Kingdom; c Department of Microbiology, Tumor and Cellbiology, Karolinska Institutet, Stockholm, Sweden, and Department of Clinical Microbiology, Karolinska University Hospital, Solna, Sweden

## Abstract

Inflammation is a crucial part of innate immune responses but, if imbalanced, can lead to serious clinical conditions or even death. Cytokines regulate inflammation, and studies report their impact on clinical outcome. However, host and pathogen genetic backgrounds influence cytokine production, making it difficult to evaluate which inflammatory profiles (if any) relate to improved prognosis. Streptococcus pneumoniae is a common human pathogen associated with asymptomatic nasopharyngeal carriage. Infrequently, it can lead to a wide range of diseases with high morbidity and mortality rates. Studies show that both pneumococcal serotype and host genetic background affect the development of disease and contribute to variation in inflammatory responses. In this study, we investigated the impact of the host and pneumococcal genetic backgrounds on pulmonary cytokine responses and their relationship to animal survival. Two inbred mouse strains, BALB/c and CBA/Ca, were infected with 10 pneumococcal strains, and the concentrations of six pulmonary cytokines were measured at 6 h and 24 h postinfection. Collected data were analyzed by principal-component analysis to identify whether there is any pattern in the observed cytokine variation. Our results show that host-pneumococcus combination was at the core of observed variation in cytokine responses, yet the resulting cytokine profile discriminated only between survivors and fatalities but not mouse or pneumococcal strains used during infection. Therefore, our results indicate that although alternative inflammatory profiles are generated during pneumococcal infection, a common pattern emerged, which determined the clinical outcome of pneumococcal infections.

## INTRODUCTION

*Streptococcus pneumoniae* is an important human pathogen associated with a wide range of diseases and high mortality and morbidity rates (1, 2). Studies show that pneumococcal serotype and host genetic background are the major factors in the development of pneumococcal disease (3–5). Pneumococcal infections are characterized by an inflammatory response whether or not the outcome is a disease. The mortality from pneumococcal disease is often linked to imbalanced inflammation, and deaths are reported despite successful pathogen clearance (6, 7). During pneumococcal disease, antibiotic treatments can exacerbate inflammation due to a rapid release of highly proinflammatory bacterial products (8). Therefore, adjuvant anti-inflammatory therapies are frequently used to support treatment of severe pneumococcal disease cases, but their clinical relevance is often disputed (7, 9, 10).

Cytokines are key players during pneumococcal infection, but which inflammatory profile constitutes a protective or pathological response or the contribution of host and pneumococcal genetics to the observed response is unclear. The inherent variation in cytokine profiles due to the host and pneumococcal genetic backgrounds is a particularly confounding factor. For example, differences seen in cytokine concentrations in BALB/c mice depend on the pneumococcal strain used but were not associated with animal mortality (11). Similarly, a panel of inbred mouse strains showed highly diverse cytokine responses to lipopolysaccharide (LPS) treatment, but again, no significant relationship with animal survival was reported (12). These observations suggest that the variation in inflammatory profiles stems from host and pathogen genetic diversity rather than clinical outcome. However, the impact of inflammatory mediators on the development of pneumococcal pneumonia is well documented (13–17), as is the relationship between genetic polymorphisms in cytokine genes and susceptibility to pneumonia, sepsis, and otitis media, which are caused by S. pneumoniae infection (18–22). It has also been shown that immunomodulation contributes to the protective effect of pneumococcal colonization and improves survival during disease (23, 24), supporting the contribution of cytokines to mortality and morbidity. However, the pleiotropic and redundant nature of cytokine responses suggests that cytokine-cytokine interactions rather than their absolute concentrations may determine the progress of pneumococcal disease.

In this study, a large number of different pneumonia phenotypes were generated to evaluate the contribution of the host and pneumococcal genetic backgrounds to the clinical outcome of pneumonia. Our results demonstrate that pneumococcal lethality and host susceptibility to infection are influenced by the host-pathogen combination. The observed variation in pneumonia phenotypes was associated with an extensive variation in the pulmonary transcriptome and pulmonary cytokine responses. Using multivariate data analysis tools, we were able to discriminate between survivors and fatalities of pneumococcal infection based on the observed pulmonary cytokine profile, therefore indicating the protective or pathological character of lung inflammation.

## MATERIALS AND METHODS

### Ethics statement.

Experiments using laboratory animals were conducted at designated University of Leicester facilities in compliance with United Kingdom Home Office license PPL 60/4327. The study was approved by the University of Leicester Ethics Committee and adhered to United Kingdom Use of Animals Act guidance.

### Mice.

Female BALB/cOlaHsd (BALB/c) and CBA/CaOlaHsd (CBA/Ca) mice were obtained from Harlan, United Kingdom, and housed at the Division of Biomedical Services at the University of Leicester. C57BL/6J mice were obtained from Charles River and housed in animal facilities of the Department of Clinical Microbiology, Karolinska University Hospital, Solna, Sweden. Mice were kept in controlled environmental facilities and fed a fundamental diet. The experiments were performed on mice at the age of 9 to 12 weeks.

### Bacterial strains.

Ten Streptococcus pneumoniae strains were used in this study: two serotype 3 (ST3) strains (SP3-BS71 [25] and BHN35 [26]), two serotype 6B strains (BHN418 and BHN191), three serotype 19F strains (BHN100, LgtSt215, and CBR206), one serotype 4 strain (TIGR4), one serotype 2 strain (D39), and one serotype 14 strain (Sp14-BS69 [25]) (Table 1). An infectious dose of each bacterial strain was prepared as previously described (5). Briefly, pneumococcal strains were incubated overnight on blood agar base (BAB) with 5% (vol/vol) horse blood at 37°C in a CO_2_-enriched atmosphere. Sweeps of colonies were then used to inoculate Todd-Hewitt broth supplement with yeast extract (THY) (BD) until the optical density at 600 nm (OD_600_) of the suspension reached 0.2. Next, the culture was inoculated into fresh THY medium at a dilution of 1:100. The growth of the bacteria was monitored until the culture reached mid-exponential phase (OD_600_ corresponding to 1 × 10^8^ to 3 × 10^8^ CFU/ml). At this point, the culture was mixed with glycerol to a final concentration of 10% (vol/vol) and stored at −70°C until further use.

**TABLE 1:** Pneumococcal strains used in this study and their lethality in the tested inbred mouse strains BALB/c and CBA/Ca^*a*^

Pneumococcal strain	Serotype	Sequence type	Lethality type	CBA/CA mice			BALB/c mice		
				% survival	Avg survival time (h)	SEM for survival time (h)	% survival	Avg survival time (h)	SEM for survival time (h)
BS71	3	180	Lethal	10	52.8	13.3	0	28	0.0
BHN35	3	180	Lethal	0	38.4	1.6	30	104	16.1
TIGR4	4	205	Lethal	0	71.5	5.5	30	106.3	14.5
D39	2	595	Mixed	10	45.7	13.7	90	161.3	6.7
BHN418	6B	138	Mixed	30	116.2	13.3	100	168	0
BHN191	6B	138	Mixed	30	93.2	18.5	90	158.2	9.8
BS69	14	124	Nonlethal	100	168	0	100	168	0
LgtSt215	19f	179	Nonlethal	100	168	0	100	168	0
BHN100	19f	179	Nonlethal	100	168	0	100	168	0
CBR206	19f	162	Nonlethal	100	168	0	100	168	0

a Lethality type indicates pneumococcal lethality based on mortality in the tested mouse strains, where lethal indicates mortality in both mouse strains, mixed indicates lethality in CBA/Ca mice only, and nonlethal indicates that no mortality was observed. Survival indicates the percentage of animals that survived infection. The average survival time is the mean survival time (hours) within the group when animals survived past 168 h (end of the experiment), a surrogate time that denotes survivors.

### Infection.

Mice were infected as described previously (5). Briefly, the challenge culture containing 1 × 10^8^ to 3 × 10^8^ CFU/ml was defrosted and centrifuged at 13,000 rpm, the supernatant was removed, and the pellet was resuspended in phosphate-buffered saline (PBS). An infectious dose at a concentration of 2 × 10^6^ CFU in 20 μl was administered intranasally to lightly anesthetized animals (2.5% [vol/vol] fluothane [Zeneca Pharmaceuticals, United Kingdom] over oxygen at a rate of 1.5 to 2 liters/min). Infected animals were then monitored every 6 h to 10 h for clinical signs until animal expiration, either when the animal was lethargic or, for survivors, at the end of the experiment, 168 h postinfection. A clinical score of 1 denoted a lack of clinical signs, a score of 2 indicated a slightly hunched animal, a score of 3 indicated a hunched animal with a starey coat (which refers to a dry hair coat where the hair appears to be standing on end or disturbed, indicating a lack of grooming and poor coetaneous circulation), a score of 4 indicated a severely hunched animal with a starey coat on part of the animal body, a score of 5 indicated a severely hunched animal with a starey coat over the entire body, a score of 6 indicated a slightly lethargic animal, and a score of 7 indicated a lethargic animal. A humane endpoint was set at a clinical score of 6 or 7.

### Mouse survival after pneumococcal infections.

Infected animals (10 animals per group) were monitored every 6 to 10 h for clinical signs until the animal was lethargic or until day 7 postinfection. An animal still alive at 7 days postinfection was considered a survivor, and this time point was also a surrogate for the endpoint of the experiment. The bacterial loads in blood were measured at 24 h postinfection and at the time when the animal was lethargic or at the end of the experiment for survivors. At 24 h postinfection, ∼25 to 50 μl of blood was collected from the tail vein and at the time of animal expiration by cardiac puncture under deep anesthesia (5.0% [vol/vol] fluothane [Zeneca Pharmaceuticals, United Kingdom] over oxygen at a rate of 1.5 to 2 liters/min). The animal was euthanized by cervical dislocation, and the lungs were removed, weighed, placed into 10 ml of PBS, and immediately homogenized. Pneumococcal CFU were tested in the lung homogenates on blood agar base plates (Oxoid) in serial decimal dilutions and then converted to CFU per milligram of lung tissue.

### Measurements of pulmonary cytokine levels.

Infected animals (6 mice per group) were euthanized by cervical dislocation at 6 h and 24 h postinfection, during which time no death due to infection was observed. The lungs were immediately removed and processed by using a Bio-Plex cell lysis kit (Bio-Rad) according to the manufacturer's protocols. Briefly, the lungs were immediately washed by using Bio-Plex cell wash buffer (Bio-Rad) and then transferred to 1 ml of Bio-Plex cell lysis buffer (Bio-Rad) with the complete protease inhibitor cocktail (Roche) for homogenization. The homogenate was then centrifuged for 5 min at 10,000 × *g*, and the supernatant was aliquoted into cryotubes and frozen at −80°C. The concentrations of seven cytokines were measured at each tested time point by using enzyme-linked immunosorbent assay (ELISA) Ready-Set-Go kits (eBioscience) for quantification of interleukin-1β (IL-1β), IL-6, IL-10, IL-17α, gamma interferon (IFN-γ), and tumor necrosis factor alpha (TNF-α) concentrations according to the manufacturer's protocols. The Ccl5 (RANTES) concentration was measured by using the Quantikine ELISA kit (R&D Systems). Each sample was diluted 10-fold, and cytokines were measured in technical triplicates. Three control samples with known cytokine concentrations were included on each microtiter plate. The optical density of the samples was measured by using an Infinite F50 ELISA plate reader (Tecan), and the cytokine concentration was calculated with GraphPad Prism using nonlinear regression. The cytokine concentration was than adjusted according to the lung weight and expressed as picograms per milligram of lung tissue.

### RNA extraction and microarray assay.

Mice (3 to 5 per group) were infected with four pneumococcal strains that are known to disseminate into lung and represent different virulence types: LgtSt215, which is nonlethal in both mouse strains; D39 and BHN418, with host-dependent virulence (lethal in CBA/Ca but nonlethal in BALB/c); and BS71, which is virulent in both mouse strains. The animals were sacrificed at 6 h postinfection, and their lungs were removed, cut into small cubes, immediately placed into RNAlater (Qiagen), and stored overnight at 4°C. RNA was isolated as described previously (5); briefly, the lungs were removed from RNAlater (Qiagen), mouse RNA was isolated by using an RNeasy minikit (Qiagen), and cRNA was synthesized by using an Ambion MessageAmp kit for Illumina arrays. Gene expression was measured by using Illumina Mouse WG-6_V2_0_R3 BeadChips, and the BeadChips were scanned on an Illumina BeadArray reader. The signal intensity was normalized (quintile normalization) across all arrays by using GenomeStudio (Illumina), and batch correction was performed by using dCHIP software (27).

### Analyses of microarray data.

The gene expression data were analyzed by using ArrayTrack (28), and the expected survival rate was based on the results of previous survival experiments. Briefly, three lists of differentially expressed genes (DEGs) were created by using a *t* test or analysis of variance (ANOVA) in ArrayTrack. The signal intensity ratios were compared between infected BALB/c and CBA/Ca mice (host factor), between animals expected to survive and animals expected to die of the infection (survival factor), and between animals infected with different pneumococcal strains: BHN418-infected mice versus LgtSt215-infected mice versus D39-infected mice versus BS71-infected mice (pathogen factor). To determine the activation of the pulmonary transcriptome by each pneumococcal strain, the gene expression of infected animals was compared to that of PBS-treated mice. A gene was considered differentially expressed when the *P* value was <0.05 and the fold change was ≥1.5. A group of genes was then selected to validate microarray results by using quantitative PCR (qPCR) as described previously (5).

### Statistical data analysis.

The cytokine concentrations at 6 h and 24 h postinfection were autoscaled, and their associations with animal survival and pulmonary and blood CFU were tested by using multivariate regression analysis in the PASW Statistics v18 package (IBM SPSS). Principal-component (PC) analysis (PCA) was performed by using Solo software (Eigenvector Research Incorporated). Cytokine data were orthogonally transformed into linearly uncorrelated coordinates and visualized after projection onto two-dimensional (2D) and three-dimensional (3D) plots to investigate clustering of the samples based on the expected survival type: survivors (no pneumococcal burdens were detected, and no death was observed), bacteremic survivors (no death was observed, although animals were bacteremic), survivors that developed pulmonary carriage (no death was observed, but pulmonary carriage was present), and fatalities (mortality rate of 70% to 100%).

### Microarray data accession number.

The gene expression data were deposited in the NCBI Gene Expression Omnibus (GEO) under accession no. GSE61459.

## RESULTS

### Clinical outcome of pneumococcal infection is controlled by both host and pathogen genetic backgrounds.

**FIG 1:**
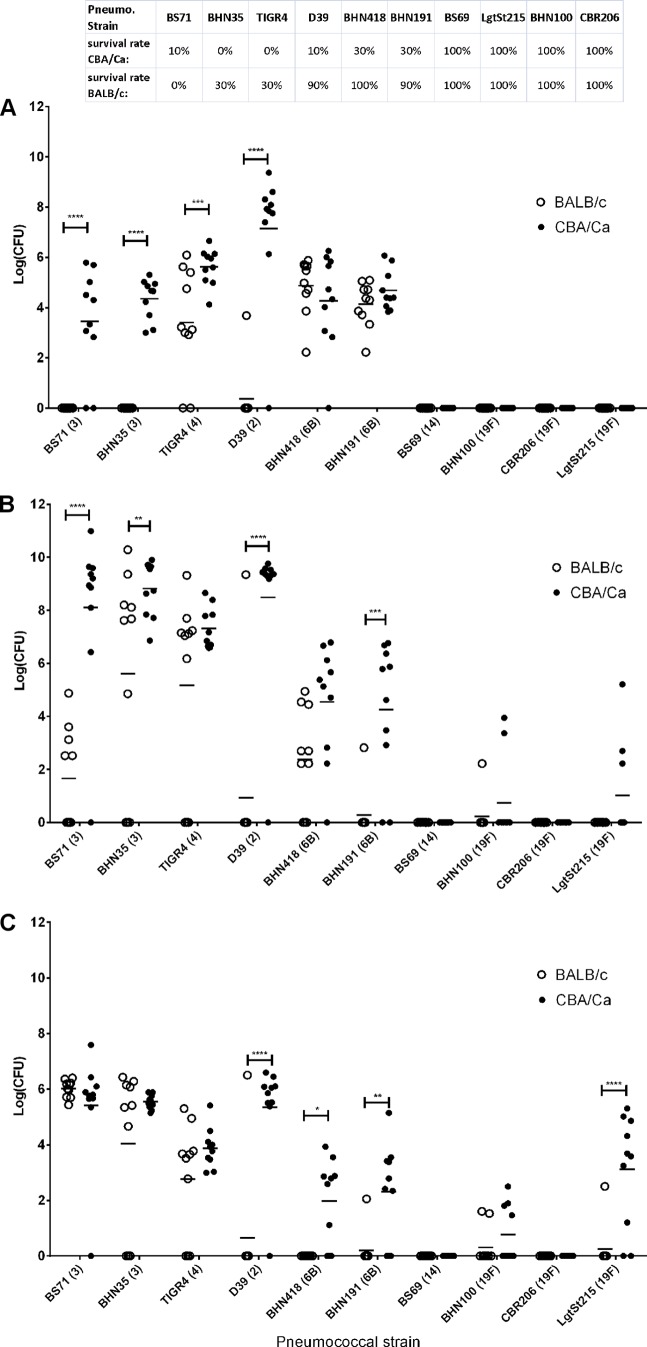
Pneumococcal burdens in tested tissues. (A) Blood at 24 h postinfection (CFU per milliliter); (B) blood at the time of animal expiration (CFU per milliliter); (C) lung at the time of animal expiration (CFU per milligram). The pneumococcal serotypes are presented in parentheses together with the strain name (*x* axis). Each experimental group consisted of 10 animals. The survival rate for each tested infection is presented at the top. For clarity, the presented statistics represent only the differences between mouse strains. *, *P* < 0.05; **, *P* < 0.001; ***, *P* < 0.0001; ****, *P* < 0.00001.

Intranasal infections of two inbred mouse strains with each of the 10 tested pneumococcal strains generated a wide range of clinical phenotypes depending on the host-pathogen combination. Although a mouse strain could generally be said to be innately resistant or susceptible to pneumococcal infection, none was found to be resistant or susceptible to all tested pneumococcal strains. Furthermore, the tested pneumococcal strains could be classified into three distinct groups based on their lethality in the tested mouse strains: (i) highly lethal regardless of the host background, such as BS71 (ST3), BHN35 (ST3), and TIGR4 (ST4); (ii) lethal dependent on the host background, such as D39 (ST2), BHN418 (ST6B), and BHN191 (ST6B); and (iii) nonlethal, such as LgtSt215 (ST19f), BHN100 (ST19F), CBR206 (ST19f), and BS69 (ST14) (Table 1). Pneumococcal strains from the same lethality group could significantly differ in their tissue dissemination and burdens, and animals with significant differences in survival could show similar CFU in tested tissues. For example, all animals reaching the humane endpoint (lethargic) had bacteria in their tissues, but surprisingly, pneumococci were also readily detected in the tissues of mice that appeared clinically normal, such as LgtSt215 (ST19f)-infected CBA/Ca mice (Fig. 1C; see also Fig. S1 in the supplemental material) or BALB/c mice infected with BHN418 (ST6B) or BHN191 (ST6B) (Fig. 1; see also Fig. S1 in the supplemental material). Indeed, the latter two strains caused similar levels of bacteremia in both inbred mouse strains (*P* > 0.05), yet mortality was observed only for CBA/Ca mice. More surprisingly, the BALB/c survivors of BHN418 (ST6B) infection still had bacteremia despite recovery and clinical signs of clearance (Fig. 1B). In contrast, at 24 h postinfection, BHN35 (ST3) and BS71 (ST3) were readily detected in the blood of CBA/Ca but not BALB/c mice (Fig. 1A), although both mouse strains displayed high mortality rates (70% to 100%).

### Pulmonary inflammation is related to host and pathogen genetic backgrounds and pulmonary CFU but is not associated with development of bacteremia at 24 h.

To begin to explain the high level of variation in pneumococcal dissemination and animal mortality following infections with different pneumococcal strains, the pulmonary concentrations of seven cytokines were measured at 6 h and 24 h postinfection. Within this time window, infected animals displayed signs of disease, but no mortality was yet observed (see Fig. S1 in the supplemental material). Following pneumococcal infections, all tested animals showed changes in the levels of at least some of the tested cytokines, but no clear pattern was identified. Therefore, pulmonary cytokine concentrations were analyzed by using a multivariate linear regression model (MLRM) to identify relationships between pulmonary cytokines and pneumonia phenotypes such as survival, lung CFU, bacteremia at 24 h, and host and pathogen genetic backgrounds. The early pulmonary cytokine profile showed significant associations with pulmonary bacterium counts at the time of animal expiration (when animals were lethargic or at 168 h postinfection) and with average survival time but not bacteremia at 24 h postinfection. Interestingly, even the cytokine profile measured at 24 h postinfection showed a poor relationship with bacteremia at 24 h (Table 2). Depending on the tested trait, different cytokines contributed to the regression model, and only two cytokines, IL-6 at 24 h postinfection and IFN-γ at 6 h postinfection, had a significant contribution to the regression model for all tested traits (Table 3).

**TABLE 2:** Relationship between pulmonary cytokine concentrations and different disease phenotypes or host and pathogen genetic backgrounds as determined by a multivariate linear regression model^*a*^

Dependent variable	Predictor								
	Cytokines at 2 time points			Cytokines at 6 h			Cytokines at 24 h		
	Pearson *R*	Adjusted *R*^2^	*P*	Pearson *R*	Adjusted *R*^2^	*P*	Pearson *R*	Adjusted *R*^2^	*P*
Lung CFU	0.903	0.791	<0.0001	0.757	0.547	<0.0001	0.822	0.655	<0.0001
Endpoint bacteremia	0.883	0.75	<0.0001	0.661	0.402	<0.0001	0.808	0.63	<0.0001
Survival	0.863	0.711	<0.0001	0.7	0.458	<0.0001	0.795	0.609	<0.0001
Bacteremia at 24 h	0.793	0.579	<0.0001	0.301	0.033	0.149	0.77	0.567	<0.0001
Pneumococcal strain	0.874	0.731	<0.0001	0.426	0.13	0.002	0.791	0.602	<0.0001
Pneumococcal serotype	0.796	0.584	<0.0001	0.601	0.321	<0.0001	0.67	0.414	<0.0001
Mouse strain	0.874	0.733	<0.0001	0.752	0.538	<0.0001	0.737	0.514	<0.0001

a Lung CFU indicate pneumococcal counts in the lung at the time of death. Endpoint bacteremia indicates pneumococcal counts in blood at the time of death. Survival indicates the average time (hours) that BALB/c or CBA/Ca mice survived after infection by each pneumococcal strain. Pneumococcal strains used were D39 (ST2), BS71 (ST3), BHN35 (ST3), TIGR4 (ST4), BHN191 (ST6B), BHN418 (ST6B), BS69 (ST14), BHN100 (ST19f), LgtSt215 (ST19f), and CBR206 (ST19f). Mouse strains used were BALB/c and CBA/Ca.

**TABLE 3:** Pulmonary cytokines that show a significant contribution in the tested multivariate linear regression models^*a*^

Cytokine	Time point (h)	Variable											
		Survival			Lung CFU			Pneumococcal strain			Mouse strain		
		Beta	T	Sig.	Beta	T	Sig.	Beta	T	Sig.	Beta	T	Sig.
IL-1β	6	0.289	2.11	0.038									
**IL-1β**	24	0.249	2.03	0.044							**−0.463**	**−3.93**	**>0.0001**
**IL-6**	6										**0.403**	**3.94**	**>0.0001**
**IL-6**	**24**	**−0.516**	**−6.71**	**>0.0001**	**0.53**	**8.12**	**>0.0001**	**−0.305**	**−4.11**	**>0.0001**	**0.372**	**5.04**	**>0.0001**
**IL-10**	6										**0.718**	**3.61**	**>0.0001**
**IL-10**	24	−0.265	−2.49	0.014				**−0.95**	**−9.27**	**>0.0001**			
IL-17a	6	0.426	2.22	0.028							0.451	2.45	0.016
IL-17a	24				−0.195	−2.19	0.031						
**IFN-γ**	6	−0.339	−2.08	0.04	0.303	2.19	0.031	**0.492**	**3.14**	**0.002**	**−0.988**	**−6.31**	**>0.0001**
IFN-γ	24												
TNF-α	6										−0.321	−2.79	0.006
TNF-α	24	−0.303	−2.39	0.019	0.238	2.20	0.03						
**Ccl5**	6				−0.302	−2.97	0.004				**−0.624**	**−5.42**	**>0.0001**
**Ccl5**	**24**	**0.259**	**3.43**	**0.001**	−0.187	−2.92	0.004				−0.168	−2.31	0.023

a Analysis was performed by using SPSS. Boldface type indicates contribution −3 > *t* > 3. Survival indicates the time (hours) that the animal survived infection. Lung CFU indicate pulmonary pneumococcal counts at the time of death. Pneumococcal strains used were D39 (ST2), BS71 (ST3), BHN35 (ST3), TIGR4 (ST4), BHN191 (ST6B), BHN418 (ST6B), BS69 (ST14), BHN100 (ST19f), LgtSt215 (ST19f), and CBR206 (ST19f). Mouse strains used were BALB/c and CBA/Ca. Beta, beta coefficient; T, *t*-statistics; Sig., significance (*P* value).

### Variation in pulmonary cytokine profiles is associated with variation in animal survival.

**FIG 2:**
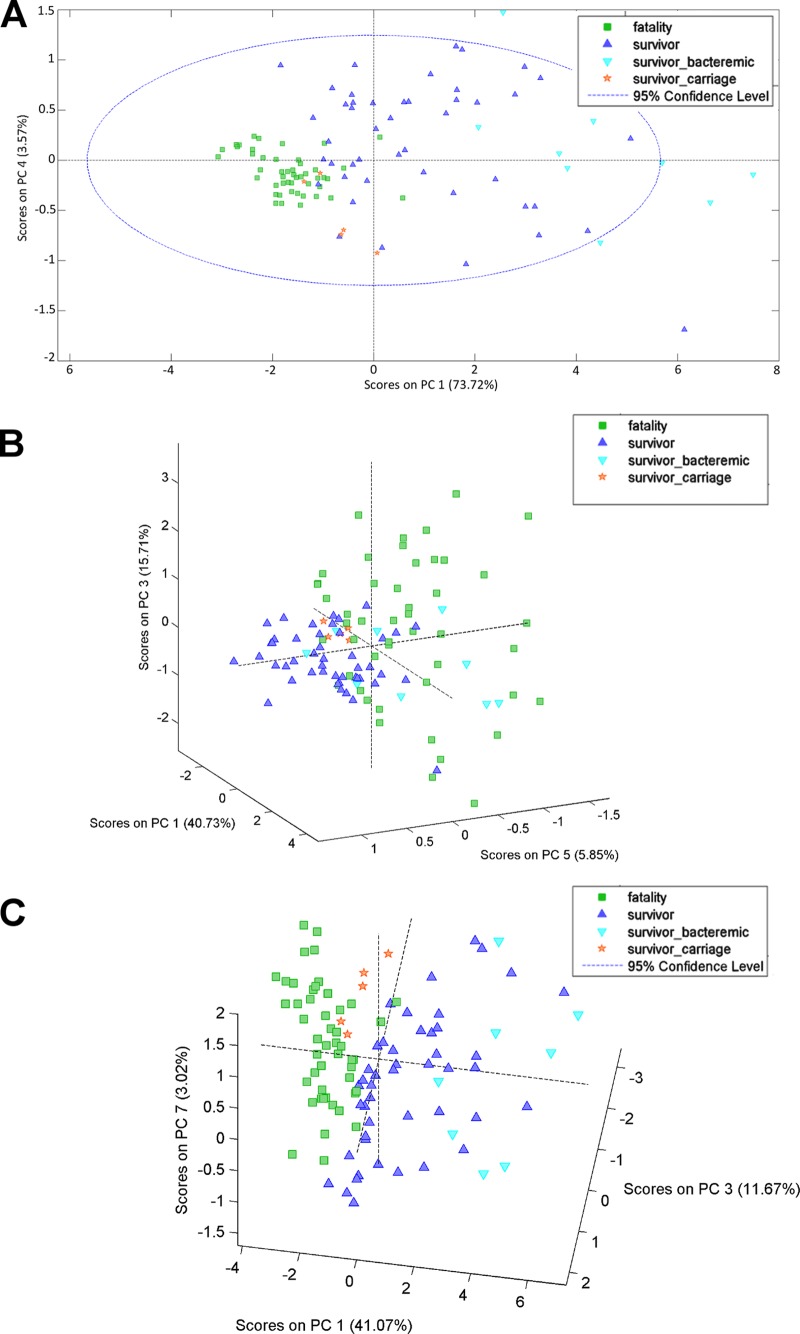
Association of mouse pulmonary inflammation with survival during pneumococcal infection. PCA plots were generated by using the cytokine concentrations measured at a single time point, 6 h (A) or 24 h (B) postinfection, or at both time points (C). The percentage of the variation captured by each PC is presented. Each data point represents one animal.

We applied another statistical approach to test the association between early pulmonary inflammation and tested traits. Cytokine concentrations were analyzed by using PCA (Table 4), and PC scores were plotted on a two- or three-dimensional diagram to visualize clustering of the samples. The PCA model based on the cytokine profile at 6 h postinfection efficiently discriminated resistant from susceptible mice using two PCs that captured 77% of the total variance (Fig. 2A). The cytokine profile at 24 h postinfection predicted animal survival based on the scores for three PCs that captured 62% of the total variance in the model, but the bacteremic survivors were incorrectly classified as fatalities (Fig. 2B). The cytokine data from both time points were then analyzed, and animal survival was correctly predicted using three PCs that captured 56% of the total variance (Fig. 2C). Indeed, the cytokine concentrations at the two time points during infection discriminated best between survivors and fatalities, with survivors that developed pulmonary carriage being placed in a separate cluster.

**TABLE 4:** PCA of pulmonary cytokine concentrations at 6 h and 24 h postinfection^*a*^

PC	Cytokines at 2 time points			Cytokines at 6 h			Cytokines at 24 h		
	% variance	Cumulative % variance	Eigen value of Cov(X)	% variance	Cumulative % variance	Eigen value of Cov(X)	% variance	Cumulative % variance	Eigen value of Cov(X)
1	41.1	41.1	5.8	73.7	73.7	5.2	40.7	40.7	2.9
2	20.5	61.5	2.9	14.2	87.9	1.0	26.9	67.7	1.9
3	11.7	73.2	1.6	5.5	93.4	0.4	15.7	83.4	1.1
4	8.5	81.7	1.2	3.6	97.0	0.3	6.8	90.2	0.5
5	5.5	87.2	0.8				5.9	96.1	0.4
6	3.4	90.6	0.5						
7	3.0	93.7	0.4						

a Percentages of the variance captured by the PC, cumulative variance, and Eigen values are presented. Cov(X), covariance of *x*.

**FIG 3:**
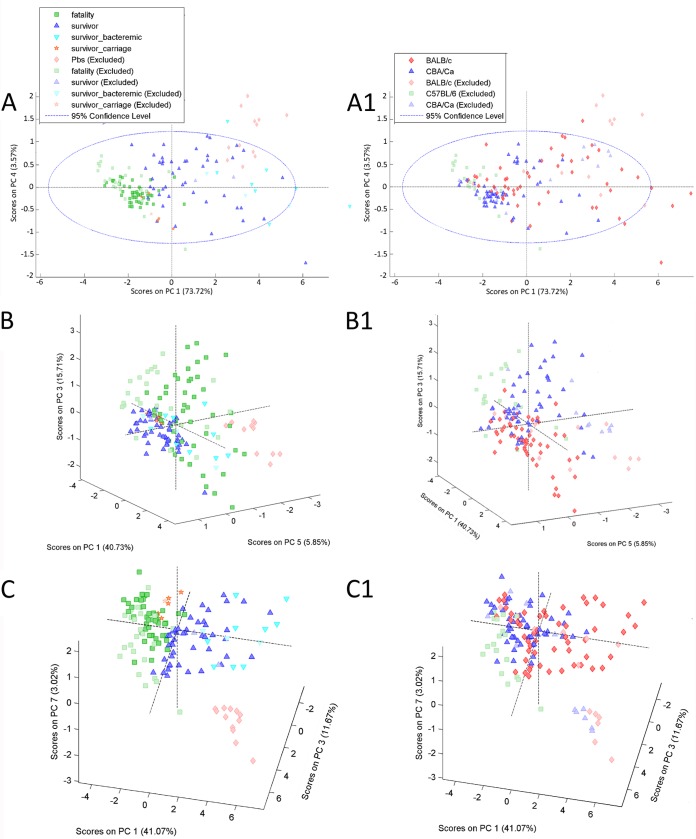
Pulmonary inflammation predicts survival of the test animals. The PCA model was validated by using lung samples of the animals previously excluded from the model. The animals from the test set (excluded) are represented by symbols with a fadeout effect. The data points for both the calibration and validation sets (fadeout colors) are presented together on the plot for better visualization of the position of the test samples within tested survival types. Each data point represents one animal. Validation using the cytokine concentrations at 6 h (A), at 24 h (B), and at both tested time points (C) is presented. The percentage of the total variation captured by the PC is shown. The genetic background of each data point is demonstrated at the right (A1 to C1).

Next, we validated the PCA model using a set of test samples, which included sham-infected animals and C57BL/6J mice infected with the D39, BS71, and BHN35 strains. The PCA models predicted correctly the expected survival of the animals in the test group, regardless of the host and pneumococcal genetic backgrounds (Fig. 3A to C).

### High mortality rates are associated with pneumococcal evasion of host molecular responses, which depend on host genetic background.

To understand the impact of the pneumococcal genetic background on the molecular responses in the lungs of tested mouse strains, we investigated changes in the pulmonary transcriptome at 6 h postinfection. The pulmonary transcriptomes of infected and PBS-treated mice were compared during infections with four pneumococcal strains, each causing different disease pathologies: LgtSt215 (ST19f) (nonlethal but readily detected in the lung), BS71 (ST3) (lethal in both mouse strains), and BHN418 (ST6B) and D39 (ST2), which have host-dependent lethality. The obtained lists of differentially expressed genes (DEGs) were then analyzed by using the DAVID online tool. Interestingly, the transcriptomic response was particularly weak in animals that showed high mortality rates (except for D39-infected CBA/Ca mice). The pulmonary transcriptomes of fatalities showed lower numbers of differentially expressed genes as well as fewer and less significant enrichment in biological themes and functionally related gene groups (Table 5). Indeed, the most severe disease caused by BS71 infection of BALB/c mice was associated with an almost complete lack of transcriptomic responses in the lung at 6 h postinfection (Table 5). However, D39-infected CBA/Ca mice did not fit the molecular pattern observed for other fatalities. Instead, their pulmonary transcriptome was similar to the one observed for nonlethal infections. However, in contrast to the survivors, D39-infected CBA/Ca mice showed significant enrichment in genes related to T-cell receptor signaling and natural killer cell-mediated cytotoxicity but not the hematopoietic cell linage pathway (Table 5) (5). Furthermore, we also took a closer look at the cytokine-cytokine receptor signaling pathway (KEGG) to identify any potential differences between fatalities and survivors of pneumococcal infections. We found that some of the receptors were differentially expressed during all or most pneumococcal infections regardless of their clinical outcome (e.g., Cxcl1, IL13RA1, Ccl19, IL1A, and IL1B), many were identified in a few tested infections (without a clear association with animal survival), and only one gene was found uniquely in nonlethal infections: CSF2RA (involved in pulmonary surfactant metabolism).

**TABLE 5:** Signaling pathways during infection with different pneumococcal strains^*a*^

Pathway	*P* value							
	BALB/c mice				CBA/Ca mice			
	D39 (ST2)	BHN418 (ST6B)	LgtSt215 (ST19f)	BS71 (ST3) (lethal)	D39 (ST2) (lethal)	BHN248 (ST6B) (lethal)	LgtSt215 (ST19f)	BS71 (ST3) (lethal)
Cytokine-cytokine receptor interaction	3.92E−07	9.10E−07	3.70E−07		2.38E−08	5.30E−04	8.20E−04	7.20E−03
Toll-like receptor signaling pathway	3.32E−04	7.70E−06	3.20E−05		1.33E−06	1.00E−03	1.60E−07	3.30E−02
Chemokine signaling pathway	9.02E−03	3.20E−04	7.80E−04		5.10E−05		2.30E−05	
MAPK signaling pathway	1.93E−05	4.30E−02	1.30E−03		1.08E−04		1.20E−02	1.10E−02
B-cell receptor signaling pathway	3.10E−03	1.30E−02	2.00E−03		4.43E−05		5.40E−04	
Hematopoietic cell lineage	1.20E−03	3.40E−03	4.40E−04			3.00E−03	3.20E−03	3.00E−04
Apoptosis	1.57E−03	4.10E−03	3.00E−03		1.09E−04	8.40E−02	4.00E−03	2.40E−02
Pathways in cancer	2.92E−05	2.60E−02	4.00E−02		6.33E−07			
Adipocytokine signaling pathway		6.20E−03	7.80E−04		1.56E−04		7.40E−04	
Jak-STAT signaling pathway	1.88E−04	1.70E−02	1.30E−02		3.02E−03		5.20E−03	
NOD-like receptor signaling pathway	8.63E−03	2.30E−02			5.88E−03		3.60E−02	
Cytosolic DNA-sensing pathway					1.05E−02		5.40E−03	
Natural killer cell-mediated cytotoxicity					9.83E−04			
Prion diseases								2.60E−02
Fc gamma R-mediated phagocytosis							8.20E−03	
Axon guidance	3.92E−03							
Complement and coagulation cascades								1.60E−02
Alzheimer's disease								4.00E−02
p53 signaling pathway	4.33E−03							
T-cell receptor signaling pathway					6.06E−03			

a Gene expression was evaluated by comparison of infected mice and PBS-treated mice. The significance (*P* value) of each pathway is presented.

### Lung innate immunity is jointly influenced by host and pathogen genetic backgrounds.

We were then interested in the impact of host-pathogen interactions on the pulmonary transcriptome and its effect on animal survival. Three lists of DEGs were obtained by comparing (i) expected survivors and expected fatalities of pneumococcal infections (survival factor), (ii) infected BALB/c and infected CBA/Ca mice (host factor), and (iii) BHN418 (ST6B)-infected mice versus LgtSt215 (ST19f)-infected mice versus D39 (ST2)-infected mice versus BS71 (ST3)-infected mice (pathogen factor).

**FIG 4:**
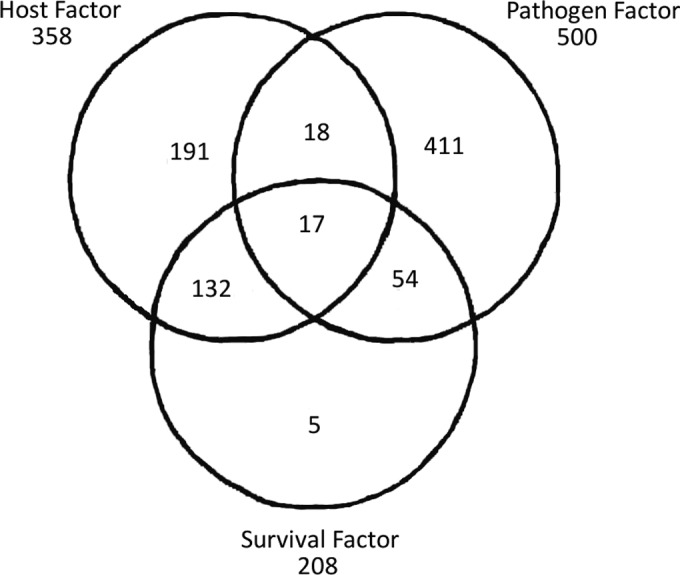
Similarities between the lists of DEGs. The lists of DEGs were generated by comparison of infected BALB/c versus infected CBA/Ca mice (host factor), mice infected with different pneumococcal strains (pathogen factor), and survivors versus fatalities of pneumococcal infections (survival factor). The number of DEGs identified in each comparison as well as the number of overlapping genes are presented.

Next, the three lists of DEGs were compared against each other to identify genes present in more than one list. We found that virtually all the DEGs associated with the survival factor were also associated with either the host or the pathogen factor (Fig. 4). Therefore, we hypothesized that the DEGs important for survival and whose expression also differed between different pneumococcal infections represented the pneumococcal contribution to differences in animal survival. Likewise, the DEGs important for survival and whose expression also differed between infected BALB/c and CBA/Ca mice represented host impact on survival. A subsequent functional analysis of the overlapping (shared) genes that were associated with the survival and pathogen factors showed significant enrichment in genes important for pathogen recognition (enriched pathways included cytosolic DNA sensing, Toll-like receptor, and NOD and mitogen-activated protein kinase [MAPK] signaling) as well as immune cell activation and migration (B- and T-cell signaling and taxis). On the other hand, genes whose expression differed between survivors and fatalities of pneumococcal infections as well as between infected BALB/c and CBA/Ca mice were related to the complement system, hematopoiesis, and cytokine signaling (Tables 6 and 7). Interestingly, expression of the genes important for antigen processing and presentation and for cell adhesion differed between BALB/c and CBA/Ca mice but not between survivors and fatalities. Similarly, expressions of the genes important for vasculature function, pathogen recognition, and chemokine and adipocytokine signaling differed between different pneumococcal infections but not between animals displaying different survival types (Tables 6 and 7).

**TABLE 6:** Comparative pathway analysis (DAVID) of DEGs^*a*^

KEGG pathway	Shared genes		Unique genes	
	Mouse *P* value	Pneumococcus *P* value	Mouse *P* value	Pneumococcus *P* value
Complement and coagulation cascades	8.00E−05			
Systemic lupus erythematosus	3.30E−03			
Hematopoietic cell lineage	0.01			
Cytokine-cytokine receptor interaction	0.02			1.00E−03
Allograft rejection	0.05			
NOD-like receptor signaling pathway		7.30E−04		
Pathways in cancer		1.90E−03		
Cytosolic DNA-sensing pathway		0.01		
B-cell receptor signaling pathway		0.02		
Toll-like receptor signaling pathway		0.03		4.90E−05
MAPK signaling pathway		0.04		
T-cell receptor signaling pathway		0.05		
Chemokine signaling pathway				1.20E−03
Leukocyte transendothelial migration				4.50E−03
Adipocytokine signaling pathway				0.03
Cell adhesion molecules			0.05	0.05
Antigen processing and presentation			0.05	
Base excision repair			0.05	

a Shared genes indicate the list of genes associated with both survival and either mouse strain or pneumococcal strain. Unique genes indicate the list of genes associated with only one of the studied factors: host (mouse) or pneumococcal strain. The significance (*P* value) of each pathway is presented.

**TABLE 7:** Comparative GO analysis (DAVID) of DEGs^*a*^

GO term	Shared genes		Unique genes	
	Mouse *P* value	Pathogen *P* value	Mouse *P* value	Pathogen *P* value
Immune response	2.30E−07		1.40E−02	1.10E−07
Immune effector process	2.70E−05			1.40E−03
Defense response	1.10E−03			2.30E−04
Positive regulation of immune response	3.80E−03			8.40E−03
Response to cytokine stimulus	9.90E−03			2.00E−03
Leukocyte-mediated immunity	8.10E−04			
Positive regulation of response to stimulus	1.80E−03			
Immunoglobulin-mediated immune response	3.20E−03			
B-cell-mediated immunity	3.50E−03			
Lymphocyte-mediated immunity	5.50E−03			
Adaptive immune response	7.20E−03			
Adaptive immune response based on somatic recombination of immune receptors built from immunoglobulin superfamily domains	7.20E−03			
Negative regulation of molecular function		2.00E−07		
Negative regulation of catalytic activity		3.10E−04		
Chemotaxis		4.20E−04		3.10E−07
Taxis		4.20E−04		3.10E−07
Cell chemotaxis		3.40E−03		
Leukocyte chemotaxis		3.40E−03		
Locomotory behavior		7.30E−03		2.20E−04
Leukocyte migration		8.50E−03		5.90E−03
Behavior		9.60E−03		5.40E−04
Negative regulation of kinase activity		5.90E−04		
Negative regulation of protein kinase activity		5.90E−04		
Negative regulation of transferase activity		6.60E−04		
Negative regulation of myeloid cell differentiation		2.10E−03		
Negative regulation of transcription factor activity		2.30E−03		
Negative regulation of cell differentiation		2.80E−03		3.80E−03
Negative regulation of DNA binding		3.20E−03		
Negative regulation of binding		4.20E−03		
Antigen processing and presentation			2.90E−03	
Response to molecule of bacterial origin				1.10E−07
Inflammatory response				1.40E−06
Response to lipopolysaccharide				2.90E−06
Vasculature development				5.50E−06
Response to wounding				7.10E−06
Regulation of cell proliferation				1.40E−05
Angiogenesis				1.60E−05
Response to organic substance				1.60E−05
Blood vessel development				1.80E−05
Positive regulation of cell differentiation				4.30E−05
Positive regulation of immune system process				4.90E−05
Positive regulation of developmental process				7.30E−05
Response to bacterium				7.40E−05
Regulation of tumor necrosis factor production				7.90E−05

a Shared genes indicate the list of genes associated with both survival and either mouse strain or pneumococcal strain. Unique genes indicate the list of genes associated with only one of the studied factors: host (mouse) or pneumococcal strain. The significance (*P* value) of each GO term is presented.

## DISCUSSION

Streptococcus pneumoniae (pneumococcus) is a common commensal of the human nasopharynx and a cause of a high morbidity and mortality rates. Pneumococcal infections characterize diverse inflammatory responses influenced by host and pathogen genetic backgrounds. Previous studies also demonstrate that cytokines affect the clinical outcome of pneumococcal infection (16–18), and immunomodulation has a beneficial effect on the survival of susceptible animals (24). Unfortunately, these results are largely derived from “one-host, one-pathogen” models and therefore do not provide information on how host and pathogen genetic variations affect inflammation and clinical outcome. To improve management of pneumococcal disease, it is essential to better understand the relationship among cytokines, host and pathogen genetic diversity, and mortality from pneumonia.

In our study, we selected two inbred mouse strains, BALB/c and CBA/Ca (4, 5), and tested their susceptibility to infection with 10 pneumococcal strains representing different pneumococcal serotypes. This approach generated a wide range of pneumonia phenotypes, enabling comprehensive analysis of host-pathogen interactions and mortality from pneumonia. We determined that the variation in disease pathology, host susceptibility, and pneumococcal virulence strongly depended on the host-pathogen combination. To better understand the underlying biological mechanism of the observed differences, we investigated the host pulmonary transcriptome and pulmonary cytokine production. Our results show that most of the fatalities of pneumococcal infection displayed an attenuated molecular response in the lung, with the most lethal pneumococcal strains also being the poorest activators of the pulmonary transcriptome. However, this was not a universal pattern, since infection of CBA/Ca mice with pneumococcal strain D39 (ST2), which resulted in a high mortality rate, was associated with significant changes in the pulmonary transcriptome similar to those observed in survivors. The noted exception was a significantly higher enrichment of genes involved in the regulation of T-cell populations in D39-infected CBA/Ca mice. Interestingly, despite the observed evasion of host immunity during lethal pneumococcal infections, we still detected significant changes in the pulmonary cytokine profile in all tested pneumococcal infections except for the BS71 (ST3) strain at 6 h postinfection. However, the observed cytokine responses significantly differed between tested pneumococcus-host combinations and during the course of the disease. Therefore, to identify a biologically relevant pattern(s), the obtained cytokine data were analyzed by multivariate data analysis.

Multivariate regression analysis identified two cytokines, IL-6 and IFN-γ, that significantly contributed to all tested regression models (host, pathogen, lung CFU, or survival) but only at a single time point during infection (at 24 h and 6 h postinfection for IL-6 and IFN-γ, respectively). The role of IL-6 and IFN-γ in pneumonia was previously described (22, 29), yet it remains unclear how they affect survival. For example, significant reductions in disease severity and pneumococcal blood invasion were observed during serotype 3 infection of mice deficient in CXCR3, a common receptor for IFN-γ-inducible chemokines such as Cxcl9, Cxcl10, and Cxcl11 (30). The role of IFN-γ during pneumococcal infection was also demonstrated in another study (29), which showed that IFN-γ-deficient mice exhibit significantly lower pulmonary pneumococcal counts, supporting the role of cytokines in the regulation of pneumococcal growth. However, the observed reduction in pneumococcal counts did not improve animal survival, therefore suggesting that changes in pneumococcal tissue burdens did not translate to a modification of the clinical outcome (29). Indeed, this conclusion was also supported by our results, which showed significant differences in pneumococcal tissue burdens in animals with similar survival rates, while no differences in CFU were observed for survivors and fatalities for some of the tested pneumococcal infections. Furthermore, in our study, infection with a serotype 3 strain did not activate IFN-γ production. We even observed a decrease in the level of pulmonary IFN-γ detected in PBS-treated mice. Subsequently, there was no expression of the IFN-γ-inducible chemokines in both BALB/c and CBA/Ca mice at 6 h postinfection. However, the absence of pulmonary IFN-γ at 6 h post-BS71 (ST3) infection was not associated with lower pneumococcal numbers or reduced disease severity in our study. Quite the opposite, both tested mouse strains exhibited high mortality rates and high pulmonary CFU. We also observed differences between BALB/c and CBA/Ca mice in serotype 3 blood invasion, with BALB/c blood being largely sterile and CBA/Ca mice developing high-level septicemia, indicating that the absence of IFN-γ did not influence BS71 (ST3) blood dissemination. Interestingly, IFN-γ is associated with T-cell populations, and our gene expression analysis showed that genes associated with T-cell functioning were significantly enriched in the pulmonary transcriptome of CBA/Ca mice during lethal infection with D39 (ST2) but not in the pulmonary transcriptome of survivors.

We then hypothesized that the cytokine profile rather than a single inflammatory mediator is a hallmark of a beneficial or pathological inflammatory process. To test this hypothesis, we used principal-component analysis to investigate clustering of the samples based on their inflammatory profile. Using this approach, we achieved good separation of tested samples according to their survival type but not according to the mouse or pneumococcal strain used in the infection. The pulmonary cytokine profile at 6 h postinfection discriminated better between survivors and fatalities than did the cytokine profile detected 18 h later, at the 24-h time point. The cytokine profile at 24 h misclassified the bacteremic survivors within the susceptible group. However, when cytokine concentrations at two time points were analyzed, the discrimination between survivors and fatalities improved. Although the PCA model was calibrated based on the cytokine concentrations in CBA/Ca and BALB/c mice, it was successfully used to predict the survival type of another inbred mouse strain, C57BL/6J, after pneumococcal infection. This result strongly supported the conclusion that the cytokine profile reflects protective or pathological inflammatory responses.

The PCA model was previously applied in a study of tuberculosis to determine the association of pulmonary inflammation with patient ethnicity or Mycobacterium tuberculosis lineage (31). That study went on to demonstrate that pulmonary inflammation was associated with patient ethnicity but not Mycobacterium tuberculosis lineage (31). Similarly, in our study, we observed that the cytokine profile was not associated with the pneumococcal strain, but instead, we showed that it was associated with animal survival. Although the relationship between pulmonary inflammation and clinical outcome was not investigated and all patients received antimicrobial therapy in the above-mentioned tuberculosis study, the differences in disease progress, response to the antibiotic, and M. tuberculosis clearance were reported to be associated with patient ethnicity. This observation indicates that ethnicity and clinical outcome were strongly correlated.

The results of this study demonstrate that host-pneumococcus interactions are responsible for the observed variations in pneumonia phenotypes, host susceptibility, and pneumococcal virulence. Despite the observed variation in pulmonary inflammation, the generated cytokine profiles display either beneficial or pathological characteristics. We also propose that the effects of a single inflammatory mediator on animal survival may depend on the inflammatory context and therefore might not be suitable to reliably predict clinical outcomes of pneumococcal infection.

## Supplementary Material

Supplemental material
