# Supplementary material for: Variation in Inflammatory Response during Pneumococcal Infection Is Influenced by Host-Pathogen Interactions but Associated with Animal Survival
Source: Infect Immun. 2016 Mar 24;84(4):894–905. doi: 10.1128/IAI.01057-15 (PMC4807497; doi:10.1128/IAI.01057-15)
Supplement: Supplemental material [file supp_84_4_894__index.html]

Supplemental material 

# Variation in Inflammatory Response during Pneumococcal Infection Is Influenced by Host-Pathogen Interactions but Associated with Animal Survival

## Supplemental material

- Supplemental file 1 -

  Fig. S1. Clinical scores for BALB/c and CBA/Ca mice after pneumococcal infections.

  PDF, 448K
- Supplemental file 2 -

  Table S1. Mean cytokine concentration for each mouse strain after infection with 1 of the 10 pneumococcal strains.

  XLSX, 21K
