# Supplementary material for: Variation in Inflammatory Response during Pneumococcal Infection Is Influenced by Host-Pathogen Interactions but Associated with Animal Survival
Source: Infect Immun. 2016 Mar 24;84(4):894–905. doi: 10.1128/IAI.01057-15 (PMC4807497; doi:10.1128/IAI.01057-15)
Supplement: Supplemental material [file IAI.01057-15_zii999091629so1.pdf]

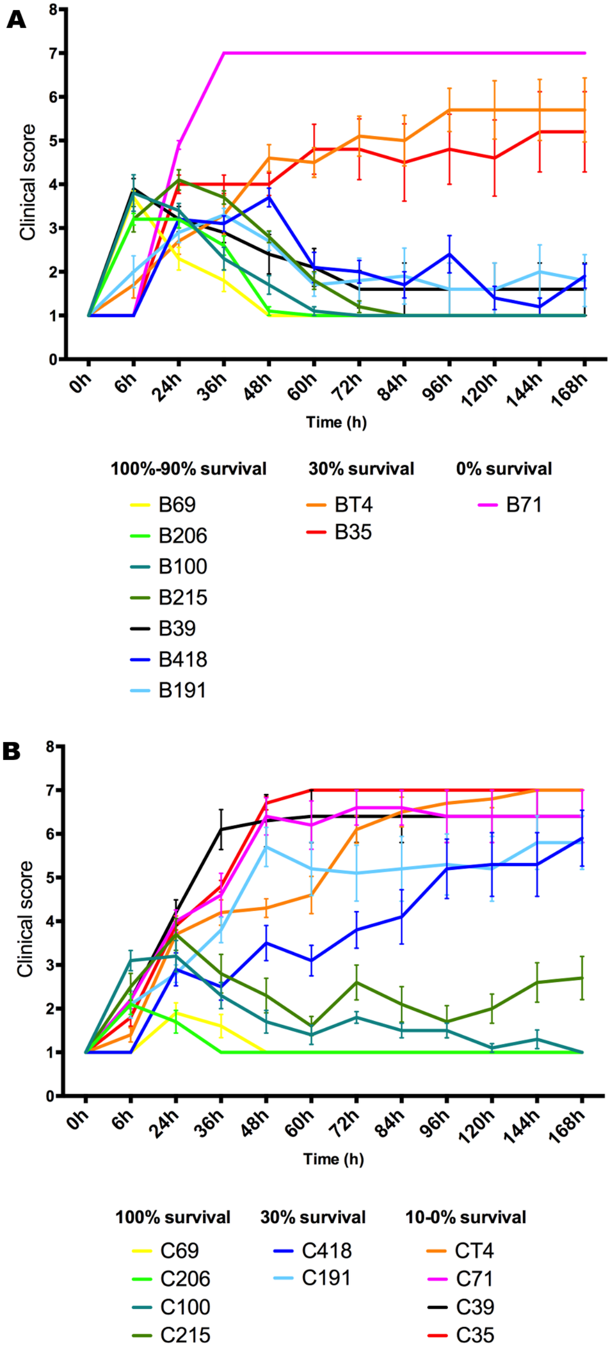

FigS1. Clinical scores for BALB/c (A) and CBA/Ca (B) after pneumococcal infections. Each experimental group is described by the letter that denotes mouse strain "B" for BALB/c or "C" for CBA/Ca and a number that denotes pneumococcal strain: "71" for BS71, "35" for BHN35, "T4" for TIGR4, "39" for D39, "418" for BHN418, "191" for BHN191, "69" for BS69, "215" for LgtSt215, "100" for BHN100 and "206" for CBR206. Clinical score of 1 denotes normal and of 7 lethargic animal. Score of 7 continue to be assigned to the expired animal until the end of the experiment (168h) (detailed explanation in the Methods section). The error bar represents standard error of the mean (SEM)
